# *Tityus serrulatus* (Scorpion): From the Crude Venom to the Construction of Synthetic Peptides and Their Possible Therapeutic Application Against *Toxoplasma gondii* Infection

**DOI:** 10.3389/fcimb.2021.706618

**Published:** 2021-07-20

**Authors:** Diego Rodney Rodrigues de Assis, Pollyana Maria de Oliveira Pimentel, Pablo Victor Mendes dos Reis, Rayane Aparecida Nonato Rabelo, Ricardo Wagner Almeida Vitor, Marta do Nascimento Cordeiro, Liza Figueiredo Felicori, Carlos Delfin Chávez Olórtegui, Jarbas Magalhães Resende, Mauro Martins Teixeira, Márcia Helena Borges, Maria Elena de Lima, Adriano Monteiro de Castro Pimenta, Fabiana Simão Machado

**Affiliations:** ^1^ Department of Biochemistry and Immunology, Instituto de Ciências Biológicas, Universidade Federal de Minas Gerais, Belo Horizonte, Brazil; ^2^ Department of Parasitology, Instituto de Ciências Biológicas, Universidade Federal de Minas Gerais, Belo Horizonte, Brazil; ^3^ Fundação Ezequiel Dias-FUNED, Minas Gerais, Belo Horizonte, Brazil; ^4^ Department of Chemistry, Instituto de Ciências Exatas, Universidade Federal de Minas Gerais, Belo Horizonte, Brazil; ^5^ Faculdade Santa Casa de Belo Horizonte: Programa de Pós Graduação em Medicina-Biomedicina, Belo Horizonte, Brazil; ^6^ Program in Health Sciences: Infectious Diseases and Tropical Medicine, Faculdade de Medicina, Universidade Federal de Minas Gerais, Belo Horizonte, Brazil

**Keywords:** *Toxoplasma gondii*, toxoplasmosis, *Tityus serrulatus* venom, immunoregulation, synthetic peptides, treatment

## Abstract

Toxoplasmosis, caused by *Toxoplasma gondii*, is a major public concern owing to its neurotropic nature and high morbidity and mortality rates in immunocompromised patients and newborns. Current treatment for this disease is inefficient and produces side effects. Inflammatory mediators produced during *T. gondii* infection (e.g., cytokines and nitric oxide) are crucial in controlling parasite replication. In this context, *Tityus serrulatus* venom (TsV) induces the production of inflammatory mediators by immune cells. Thus, this study aimed to isolate and identify the components of TsV with potential anti-*T. gondii* activity. TsV was extracted from scorpions and lyophilized or loaded onto a column to obtain its fractions. TsV subfractions were obtained using chromatography, and its amino acid sequence was identified and applied to peptide design using bioinformatics tools. The C57BL/6 mice and their harvested macrophages were used to test the anti-*Toxoplasma* activity of TsV components and peptides. TsV and its fraction F6 attenuated the replication of tachyzoites in macrophages and induced nitric oxide and cytokine (IL-12, TNF, and IL-6) production by infected cells, without host cell toxicity. Moreover, Su6-B toxin, a subfraction of F6, demonstrated anti-*T. gondii* activity. The partially elucidated and characterized amino acid sequence of Sub6-B demonstrated 93% similarity with *T. serrulatus* 2 toxin (Ts2). Ts2 mimetic peptides (“Pep1,” “Pep2a,” and “Pep2b”) were designed and synthesized. Pep1 and Pep2a, but not Pep2b, reduced the replication of tachyzoites in macrophages. *In vivo*, treatment of *T. gondii*-infected mice with Pep1, Pep2a, or Pep2b decreased the number of cerebral cysts and did not induce hepatotoxicity in the animals. Taken together, our data show promising immunomodulatory and antiparasitic activity of TsV that could be explored and applied in future therapies for treating infectious parasitic diseases such as toxoplasmosis.

## Introduction

Toxoplasmosis, caused by the parasite *Toxoplasma gondii*, affects one-third of the global population. This infection is most often not characterized as a lethal disease in humans, but it can be fatal in immunocompromised individuals, such as HIV/AIDS, transplanted, or cancer patients, and pregnant women (fetus). Importantly, there is increasing concern about this infection in immunocompetent individuals owing to the neurotropic nature of this parasite, associated development of mental and behavioral disorders, ocular toxoplasmosis causing focal necrotizing retinochoroiditis, and potential myocarditis or polymyositis development (Montoya and Liesenfeld, 2004; Weiss and Dubey, 2009).

The main line of defense against *T. gondii* infection involves cells of the innate and acquired immune responses, including dendritic cells, natural killer cells, macrophages (MOs), and lymphocytes. These cells produce inflammatory mediators, such as interleukin (IL)-12, tumor necrosis factor (TNF), and interferon-gamma (IFN-γ), triggering the production of microbicidal/microbiostatic molecules (e.g., nitric oxide; NO and reactive oxygen species; ROS) and indoleamine 2,3-dioxygenase enzyme activity that limits parasite replication, highlighting the central role of MOs in the control of parasite growth and dissemination (Denkers and Gazzinelli, 1998; Sasai et al., 2018).

New strategies/drugs for toxoplasmosis treatment are working slowly owing to the complexity of the parasite, which is protected from external agents in the host cell, limiting the action of the drugs. Additionally, drugs that are used currently in clinics are not effective in eradicating the latent bradyzoite stage of the parasite, produce considerable side effects (Alday and Doggett, 2017), and present reduction in effectiveness possibly due to the appearance of drug-resistant strains (Montazeri et al., 2018), which underlines the need for new therapeutic approaches against this infection.

Modulation of the immune system is characterized as a tool for the treatment of some diseases (van Kasteren et al., 2018), including toxoplasmosis, where *T. gondii* can restrict immune cell activation, inhibit the production of pro-inflammatory mediators, and stimulating the production of anti-inflammatory/resolving mediators, favoring its establishment in the host organism, thereby leading to chronic infection (Lüder et al., 2001; Bannenberg et al., 2004; Machado et al., 2006; Laliberté and Carruthers, 2008; Hunter and Sibley, 2012).

*Tityus serrulatus* (Brazilian yellow scorpion) venom (TsV), is composed of a mixture rich in proteins, peptides, and minor components, such as salts, hormones, amino acids, and sugars (Cologna et al., 2009), and presents immunomodulatory activities. TsV induces a pro-inflammatory response, stimulating cells, such as MOs, to produce TNF/IL-12/IFN-γ/NO/ROS. Moreover, TsV stimulation increases the phagocytic capacity of MOs and activates T lymphocytes (Petricevich et al., 2008; Zoccal et al., 2011; Casella-Martins et al., 2015), which are stimulators of immunity.

Several natural compounds have been found from diverse sources, including scorpion venoms, which are candidates for the development of new drugs, for example, more effective antitumor, antimicrobial and antichagasic agents (Ortiz et al., 2015; Lichota and Gwozdzinski, 2018; Herzig et al., 2020; Pimentel et al., 2021). This study aimed to explore the immunomodulatory and antiparasitic activities of TsV and its components as potential alternative chemotherapies against *T. gondii* infection.

## Methods

### Animals and Ethics Statement

C57Bl/6 female mice (8–10 weeks old) were obtained from the animal care facilities of Universidade Federal de Minas Gerais (UFMG), Minas Gerais, Brazil. This research was conducted according to the Brazilian Guidelines on Animal Work and the Guide for the Care and Use of Laboratory Animals of the National Institutes of Health (NIH). The UFMG Animal Ethics Committee (CEUA) approved all experiments and procedures (Permit Number 449/2015). The research activity was registered at *Sistema Nacional de Gestão do Patrimônio Genético* (SIsGEN) (numbers AF3F637, A5685EA, and A885487) and at *Conselho Nacional de Desenvolvimento Científico e Tecnológico/Conselho de Gestão do Patrimônio Genético* (CNPq/CGEN/MMA; authorization number 010815/2015-5).

### TsV Production

TsV was extracted from scorpions at the Ezequiel Dias Foundation (FUNED) serpentarium. For assays, lyophilized TsV was resuspended in sterile water for injection (SAMTEC^®^, Ribeirão Preto, SP, BR), filtered (membranes/0.22 µ; MILLEX^®^, Merck-Millipore), and stored at -20°C.

### Fractionation of TsV (F1 to F7)

To obtain F1, F2, F3, F4, F5, F6, and F7 fractions, TsV was separated by a combination of gel filtration, ion-exchange and reverse phase chromatography. Initially, 250 mg of the sample was solubilized in 2 mL of ammonium bicarbonate buffer (0.5 M/pH 7.4) and centrifuged at 5000 × *g* for 20 min. The supernatant was collected and loaded onto a sephadex G-50 (Superfine) column, pre-equilibrated with the aforementioned buffer. Peaks (F1 – F7 fractions) were eluted at a flow rate of 0.5 mL/min based on protein detection at 280 nm, followed by lyophilization and storage at -20°C until use.

### Subfractionation of F6 (Obtention of Sub-A and Sub-B)

To obtain the subfractions 6A and 6B, the peak corresponding to fraction F6 was subjected to HiTrap-SP HP cation exchange chromatography with a gradient of 0.01 M sodium acetate buffer (pH 4.7, solution A) and solution B (0.01 M sodium acetate buffer, pH 4.7, 0.5 M NaCl) at flow rate 1mL/min, resulting in 17 peaks detected at 214 nm. Each fractionation peak was subjected to reverse phase chromatography (Vydac C18 column, flow rate 1 mL/min) in a gradient system containing 0.1% v/v trifluoroacetic acid solution in water (Solution A) and 0.1% v/v trifluoroacetic acid solution in acetonitrile (Solution B) to obtain the homogeneous molecules, followed by MALDI-TOF mass spectrometry analysis. To further characterize the molecules (peptides), their partial sequence was elucidated by Edman degradation using a Shimadzu PPS-21A automated protein sequencer.

### Toxin Identification and Peptide Design

The Sub6-B molecule was sequenced (*de novo* sequencing method) and the following partial amino acid composition (~47%) was found: (KEGYAMDHEGCCFSCFIGPAGF CDGYCCC…); to identify it, a protein BLAST search was performed using the default parameters against the non-redundant (nr) database (Altschul et al., 1990). The alignment was visualized using Jalview 2.11.0t (Waterhouse et al., 2009). First, a three-dimensional model of the identified TsV alpha-mammal toxin Ts2 (Uniprot: P68410.1) was obtained *via* homology modeling using the software package Modeller 9v16 (Webb and Sali, 2016). To build the model, the X-ray structure of Neurotoxin (Ts1) (PDB accession code: 1B7D) from TsV (Polikarpov et al., 1999) was used as a template. Modeller script salign was used for the alignment, QMEAN for model evaluation (Benkert et al., 2008), and image rendering was performed using the Pymol package (DeLano, 2002). The bioinformatics tool PEPOP (Demolombe et al., 2019) was used to design peptides representing potential discontinuous epitopes from Ts2. The optimized nearest neighbor path (“ONN”) method was used to build (extend) the peptide primary structure. Thus, two peptides carrying an acetylated (CH_3_CO) N-terminus and C-terminal carboxamide (NH_2_) were generally named “Pep1” and “Pep2a”; a third peptide sequence, named “Pep2b”, was designed by removing the N-terminal acetyl group of Pep2a.

### Peptide Synthesis

The C-terminal amidated peptides were synthesized on a Rink amide resin using the Fmoc (9-fluorenylmethyloxycarbonyl) strategy as described elsewhere (Gomes et al., 2018). N-terminal acetylation of Pep1 and Pep2a was promoted by the reaction of the respective peptidyl-resins with acetic anhydride before the peptide-resin cleavage. Peptide quality control was evaluated by mass spectrometry (MALDI-ToF/ToF; Autoflex III smartbeam Bruker Daltonics, Billerica, USA) and by both MS and MS/MS (peptide fragmentation). Briefly, a matrix (CHCA; 1:1, v/v) was used to crystallize peptide samples on MTP AnchorChip 600/384 plates (Bruker Daltonics, Billerica, MA, USA). Autoflex III was operated in the positive reflector mode, using external calibration. The obtained spectra were analyzed by using FlexAnalysis 3.1 (Bruker Daltonics, Billerica, MA, USA). The lyophilized peptides were diluted in phosphate-buffered saline solution (*1×* PBS), filtered, and maintained at -20°C until use.

### Macrophage Culture and *T. gondii* Infection

Mice were injected with 2 mL of sterile sodium thioglycolate solution at 3% v/v (DifcoTM^®^ fluid), and after 72 h, MOs were harvested from the peritoneal cavity by washing with sterile and cold *1×* PBS. The cells were centrifuged (290 × *g*, 10 min), counted, plated (2 x10^6^ cells/well) onto culture plates (24 wells; TPP^®^), placed or not a round coverslip onto bottom of each of the wells, and maintained in an incubator at 37°C and 5% CO_2_ in the presence of Roswell Park Memorial Institute 1640 culture medium (RPMI, Gibco, Grand Island, NY, USA) supplemented (RPMIc) with 5% v/v fetal bovine serum (Cultilab, Campinas, SP, BR), 1% v/v L-glutamine (Gibco), and 1% v/v penicillin/streptomycin (Gibco) for 3 h, followed by a wash, addition of RPMIc, and overnight incubation for subsequent infection/stimulation. The tachyzoite forms of *T. gondii* (RH strain) were cultured in the *kidney epithelial cell line LLCMK2 (Macaca mulatta*– “Rhesus”) in the presence of RPMIc. The parasites were centrifuged twice (50 × *g* for 5 min, and then 2438 × *g* for 10 min), counted, and used to infect the MOs (at a 1:1 parasite/host cell ratio), for 2 h, followed by a wash (RPMIc). The uninfected and infected MOs were stimulated or not with TsV (100, 200 and 400µg/mL) or TsV fractions 6 and 7 (100 or 50 μg/mL, respectively) or Sub6-A, Sub6-B (100 and 50 μg/mL) or peptides (25, 50 and 100 μg/mL) for 4, 24 or 48 h. Intracellular tachyzoites counting was determined by light microscopy in macrophages/coverslips fixed and stained with hematoxylin/eosin method (*Panótico Rápido*; LB Laborclin, Pinhais, PR, BR), and mounted onto microscope slides (triplicates) using Entellan mounting medium (Merck, KGaA, Darmstadt, Germany). Total 300 cells were counted for each coverslip discriminating between uninfected and infected cells. Moreover, at 24 and 48 h after stimulation, supernatants were collected for subsequent viability assay and nitric oxide and cytokine quantification. For parasite pre-stimulation experiments, the tachyzoites were incubated in the presence of TsV or F6, both at 100 µg/mL, or RPMIc alone for 1 h, followed by washing with RPMIc, and used for MO infection (as described above).

### Cell Viability Assay

Peritoneal macrophages were obtained as described above, and plated (2 × 10^6^ cells/well into 24-well culture plate), and incubated for 3 h (37°C, 5% CO_2_). Then, the cells were washed with RPMIc and incubated overnight. Subsequently, the cells were first washed with RPMIc medium either treated with 2% v/v triton X100 (Sigma-Aldrich Corp., St. Louis, MO, USA) (death cell positive control), TsV (100 µg/mL), F6 (100 µg/mL), or F7 (50 µg/mL). At 48 h after incubation, cell viability was performed using the colorimetric method MTT (3-(4,5-Dimethyl-2-thiazolyl)-2,5-diphenyl-2H-tetrazolium bromide) in adherent cells, as previously described (Plumb, 2004). The reading was performed using a spectrophotometer (Bio Tek- Elx800) at 490 nm. The percentage of viable cells was calculated compared to the average ratio of control cells (incubated with RPMIc only).

### Measurement of Nitric Oxide and Cytokines

Macrophages was plated, infected and/or stimulated with TsV, F6 or F7 as described above. The supernatants were harvested at 24 and 48 h after infection and/or stimulation and assayed for nitrite concentration using the Griess colorimetric method, as previously described (Green et al., 1982). Briefly, 50 μL of culture supernatant was mixed with 50 μL of Griess reagent. Absorbance at 540 nm was read using a spectrophotometer (Elx800 – Bio Tek) 10 min later. The $\text{N}{\text{O}}_{2}^{-}$ concentration was determined by reference to a standard curve of 500 μM (initial concentration) undergoing serial dilutions (factor 2). Additionally, the cytokines (IL-12p70, TNF, and IL-6) were assayed in the supernatants of the macrophage cultures at 24 and 48 h after infection and/or stimulus with TsV, F6, or F7 by enzyme-linked immunosorbent assay (ELISA) method, according to the manufacturer’s instructions (kit, R&D Systems Inc., Minneapolis, Minnesota, MN, USA). Briefly, 100 µL of monoclonal antibody specific for a particular cytokine (IL-12p70, TNF or IL-6) was pre-coated onto a 96-well microplate and incubated overnight at room temperature. Then, the plate was washed (wash buffer) and blocked by adding 300 µl/well of reagent diluent, followed incubation at room temperature for 1h. After washing, 100 µL of standards and samples (culture supernatant) were added to the wells and incubated for 2h at room temperature. After washing the unbound substances, the detection antibody (specific for the cytokine of interest; 100 µL) was added to the wells and incubated for 2h at room temperature. Following a wash, 100 µL of streptavidin-HRP solution was added and the plate was incubated for 20 min at room temperature. Then, the plate was washed and the substrate solution (100 µL) was added to the wells, incubated for 20 min at room temperature, and 50 µL of stop solution was added to the wells, and the color development was measured as optical density at 450 nm using a spectrophotometer (BioTek - Elx800). Moreover, to determine serum levels of IL-12p70 and IFN-γ, 5, 7 and 30 days after infection (dpi) and/or treatment with Pep1, Pep2a, or Pep2b, blood was collected from mice and allowed to clot at room temperature, and the sera were separated and stored at -20 °C. The sera were brought to ambient temperature and cytokines were measured by ELISA (kit, R&D Systems Inc.) as described above.

### Treatment of *T. gondii*-Infected Mice With Ts2-Derived Peptides

For *in vivo* experiments, infective *T. gondii* cysts (ME49 strain) were obtained from the brains of chronically infected mice (35 – 45 dpi). Briefly, C57Bl/6 mice were euthanized, their brains were removed and washed with sterile *1×* PBS, macerated, and homogenized with a needle and syringe in sterile *1×* PBS (1 mL). This suspension (10 µL) was placed on a glass microscope slide and the number of cysts was determined microscopically. Next, C57Bl/6 female mice, 9–10 weeks old, were intraperitoneally infected with 20 cysts/animal. Treatment with Pep1, Pep2a, or Pep2b (at a dose of 1 mg/kg, intraperitoneally) started 8 h after infection and lasted until 7 dpi, with a 24/24 h interval. The animals were monitored throughout the experiment (weight loss and survival) at 5, 7, and 30 dpi. Blood samples were collected for cytokine analysis in serum (as described above). Additionally, the presence of liver enzymes (glutamic oxaloacetic transaminase [GOT] and glutamic pyruvic transaminase [GPT]) was quantified (Bioclin^®^ kinetic assay, Belo Horizonte, MG/BR). At 30 dpi, the animals were euthanized and the brains cysts were quantified as described above.

### Statistical Analysis

Statistical analyses were performed using Prism v. 8.2.1 (GraphPad software, San Diego, CA, USA) by comparing the means ± SEM from controls to the treated samples using the Student’s t-test and one- or two-way ANOVA with the Bonferroni test. Differences were considered significant when *p* ≤ 0.05.

## Results

### TsV Induces the Production of Inflammatory Mediators by Macrophages, Reducing *T. gondii* Replication

**Figure 1:**
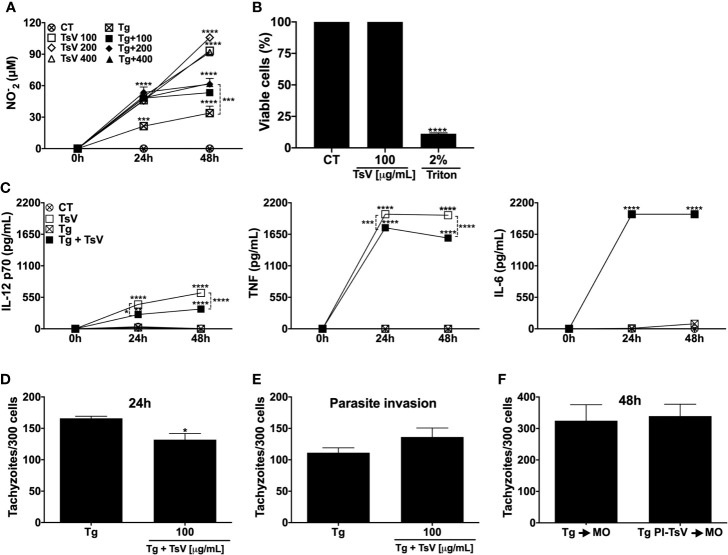
TsV induces the production of inflammatory mediators by macrophages, reducing *T. gondii* replication. Peritoneal macrophages from C57Bl/6 female mice were cultured and infected (1:1 *T. gondii*: cell ratio) and/or stimulated with TsV (100, 200, or 400 µg/mL). After 24 and 48 h, the culture supernatants were collected for nitric oxide **(A)** and IL-12p70, TNF, and IL-6 cytokine **(C)** measurements. Viability assay was performed on the adhering cells at 48 h after TsV (100 µg/mL) stimulation **(B)**. Intracellular parasite counts were performed on fixed and stained cells at 24 h **(D)** or 4 h **(E)** post-infection/stimulus. Macrophages were infected with TsV (100 µg/mL)-pre-incubated tachyzoites forms (“PI”) and 48 h after infection, the cells were fixed and stained for intracellular parasite quantification **(F)**. “CT”= control (non-stimulated cells). “Tg”= *T. gondii*-infected macrophage. Each point represents means ± SEM of one of two independent experiments, *p ≤ 0.05; ***p < 0.001; ****p < 0.0001.

First, was evaluated the effect of TsV on nitric oxide production, an important antiparasite mediator. As demonstrated in **Figure 1A**, cells incubated with TsV at different concentrations (100, 200, or 400 µg/mL) were stimulated to produce significant levels of NO. Notably, *T. gondii*-infected MOs stimulated with TsV inhibited the production of NO (**Figure 1A**). However, at 24 h, NO production remained significantly higher in the stimulated than the non-stimulated infected cells. Since there was no significant difference in the induction of NO levels among the concentrations tested, the 100 µg/mL concentration was chosen for the subsequent tests. Cell viability assay (MTT) demonstrated that TsV at 100 µg/mL was not toxic to MOs 48 h after incubation compared with the unstimulated cells (**Figure 1B**). Moreover, TsV also stimulated the production of IL-12p70, TNF, and IL-6 (the critical cytokines for an efficient immune response against *T. gondii*), at 24 and 48 h after stimulation (**Figure 1C**). As demonstrated in **Figure 1C**, *T. gondii* infection did not induce significant production levels of these pro-inflammatory cytokines by MOs, which is described as an immune suppression mechanism induced by the parasite to evade the host response. Besides, the infection resulted in decreased production of IL-12 and TNF, but not IL-6, at 24 h and 48 h. However, the TsV stimulus induced the production of higher levels of IL-12 and TNF compared with infected-unstimulated cells (**Figure 1C**). TsV anti-protozoal activity by the infected MOs was assessed within 24 h after infection and/or stimulation. **Figure 1D** shows that the TsV-stimulated cells presented a reduced number of intracellular tachyzoites compared with the unstimulated infected cells. Additionally, the venom did not change the invasion of host cells by parasites (**Figure 1E**). To examine the direct effect of TsV on the parasite, MOs were infected with tachyzoites that were previously incubated with TsV 100 µg/mL. As demonstrated in **Figure 1F**, incubation of the parasite with TsV prior to infection did not affect its replication capacity.

### The F6 Fraction From TsV Triggers the Production of Pro-Inflammatory Mediators and Induces Macrophage Anti-Protozoal Activity

**Figure 2:**
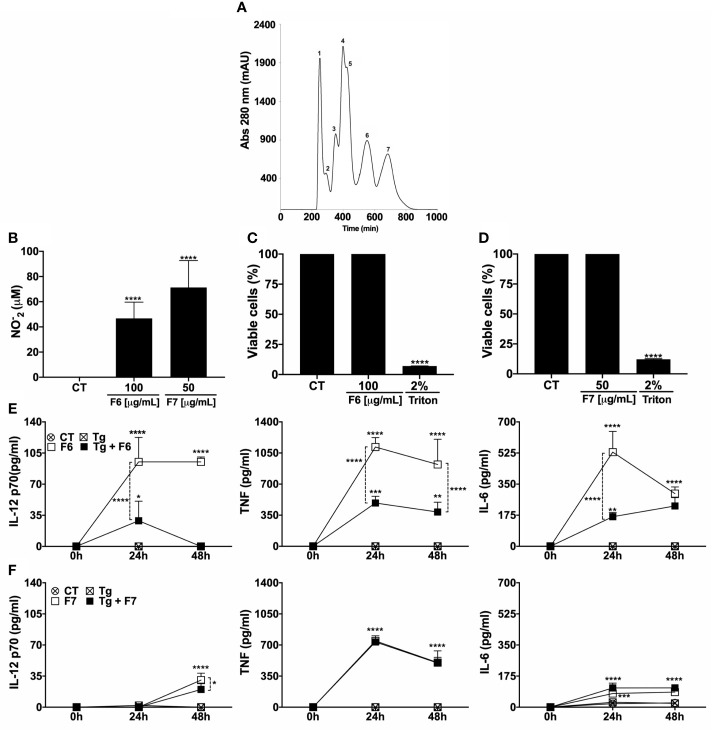
F6 fraction from TsV triggers the production of pro-inflammatory mediators and induces macrophage anti-protozoal activity. TsV was separated by gel filtration chromatography in which the components were eluted at a flow rate of 0.5 mL/min and protein detection was performed at 280 nm. The fractions obtained were listed from 1–7 **(A)** Peritoneal macrophages were cultured and stimulated with F6 or F7 fractions (100 or 50 µg/mL, respectively), and at 48 h post-stimulation the culture supernatants were collected for nitric oxide measurement **(B)**. Viability assay (MTT) was performed on the adhering cells at 48 h after F6 (100 µg/mL) **(C)** or F7 (50 µg/mL) stimulation **(D)**. The cells were infected (1:1 parasite: cell ration) and/or stimulated with F6 (100 µg/mL) or (F7 50 µg/mL) and the culture supernatants were collected at 24 and 48 h post-stimulus; **(E)** F6 and **(F)** F7, for cytokine measurements (IL-12p70, TNF, and IL-6). Each point represents means ± SEM of one of the two independent experiments, *p ≤ 0.05; **p < 0.01; ***p < 0.001; ****p < 0.0001.

Recently, our group isolated and characterized the key protein fractions present in TsV that were involved in the induction of NO in MOs (Pimentel et al), here named F1 – F7, according to the order of elution from the column (**Figure 2A**), and only F6 and F7 induced significant production of NO (Pimentel et al., 2021). Accordingly, F6 at 100 µg/mL and F7 at 50 µg/mL induced significant production of NO by MOs (**Figure 2B**). F6 at 100 µg/mL (**Figure 2C**) and F7 at 50 µg/mL (**Figure 2D**) were not toxic to MOs and both induced the production of IL-12, TNF, and IL-6 (**Figures 2E, F**). Notably, *T. gondii*-infected MOs did not produce significant levels of the analyzed cytokines (**Figures 2E, F**). *T. gondii* infection resulted in decreased production of IL-12 and TNF (24 and 48 h) and IL-6 (24 h) compared to the uninfected F6-stimulated cells. However, the levels of IL-12 (24 h), TNF (24 and 48 h), and IL-6 (24 and 48h) remained higher than that of the unstimulated infected cells (**Figure 2E**). Additionally, *T. gondii* decreased the levels of IL-12 (48h), but not TNF or IL-6 production triggered by F7. Notably, F6 was a better inducer of cytokine production than F7. In summary, our results indicate that F6 and F7, mainly F6, retain the molecules that might participate in the control of *T. gondii* replication, inducing a pro-inflammatory profile in MOs. Thus, the F6 fraction was fractionated to isolate and characterize the molecules with possible anti-*T. gondii* activity.

### Subfraction Sub6-B From F6 Induces Potent Control of *T. gondii* Replication by Macrophages

**Figure 3:**
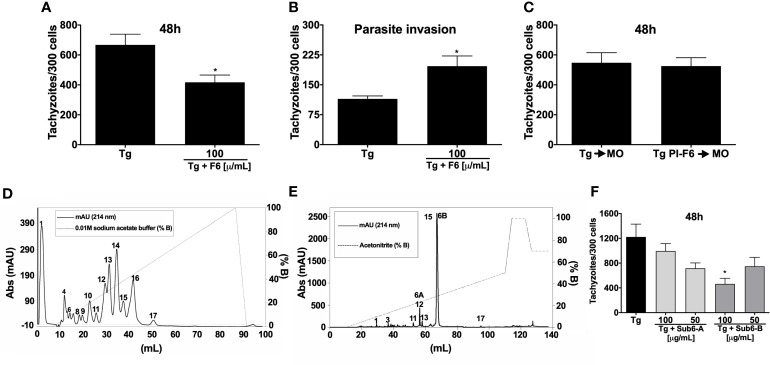
Subfraction Sub6-B from F6 induces potent control of *T. gondii* replication by macrophages. Peritoneal macrophages were cultured and infected (1:1 parasite: cell ration) and/or stimulated by F6 (100 µg/mL). Intracellular parasite counts were performed on fixed and stained cells at 48 h **(A)** or 4 h **(B)** post-infection/stimulus. Macrophages were infected with F6 (100 µg/mL)-pre-incubated tachyzoite forms (“PI”) and the intracellular parasites were quantified 48 h after infection **(C)**. Each point represents means ± SEM of one of the two independent experiments,*p ≤ 0.05. For purification of molecules, fraction F6 was subjected to cation exchange chromatography in which the components were eluted at a flow rate of 1 mL/min and peaks detected at 214 nm **(D)**, and the resulting peak 13 was subjected to reversed-phase chromatography with a flow rate of 1 mL/min and detection at 214 nm, followed by MALDI-TOF mass spectrometry and Edman degradation analyses, resulting in subfractions listed from 1–17 **(E)**. The infected-macrophages were stimulated with homogeneous molecules (toxins): Sub6-A (peak 12) or Sub6-B (peak 15) (100 or 50 µg/mL). Intracellular parasite growth was analyzed on fixed and stained cells at 48 h post-infection/stimulus **(F)**. Each point represents means ± SEM of one experiment, *p ≤ 0.05.

The potential of F6 anti-*T. gondii* activity in MOs was confirmed by the reduced number of parasites in the stimulated cells compared to the unstimulated cells (**Figure 3A**). Notably in contrast to TsV (**Figure 1E**), when F6 was simultaneously incubated with the parasites and macrophages, increased parasite invasion was observed (**Figure 3B**). Similar to TsV (**Figure 1F**), F6 pre-incubated parasites did not alter their replication capacity (**Figure 3C**). These data confirmed the promising immunomodulatory ability of the components present in F6, leading to *T. gondii* replication control. Hence, the next step was to further fractionate F6 to obtain purified toxins. For this, F6 was subjected to HiTrap-SP HP cation exchange chromatography (flow 1 mL/min, absorbance at 214 nm) with a gradient of 0.01 M sodium acetated buffer (% B), resulting in 17 new chromatography peaks that were listed according to the order of elution (peaks 1–17) (**Figure 3D**). Subsequently, peak 13, with the best activity, was subjected to reversed-phase chromatography (Vydac C18 column, flow 1 mL/min, absorbance at 214 nm) in a gradient system containing 0.1% v/v trifluoroacetic acid solution in water and 0.1% v/v trifluoroacetic acid solution in acetonitrile to isolate pure molecules, followed by MALDI-TOF mass spectrometry and Edman degradation analyses, from which 17 molecules were isolated and listed according to the order of elution (peaks 1–17) (**Figure 3E**). Because of the small amounts obtained, only two compounds, named Sub6-A and Sub6-B (related to peaks 12 and 15, respectively), were subjected to the antiparasitic activity assay in MOs. To test the antiprotozoal activity of Sub6-A and Sub6-B, infected MOs were stimulated with these compounds at 100 or 50 µg/mL. As demonstrated in **Figure 3F**, Sub6-B at 100 µg/mL was able to significantly control tachyzoite replication compared to the unstimulated and groups stimulated with the other subfractions.

### Characterization of Sub6-B Subfraction and Mimetic Peptide Production

**Figure 4:**
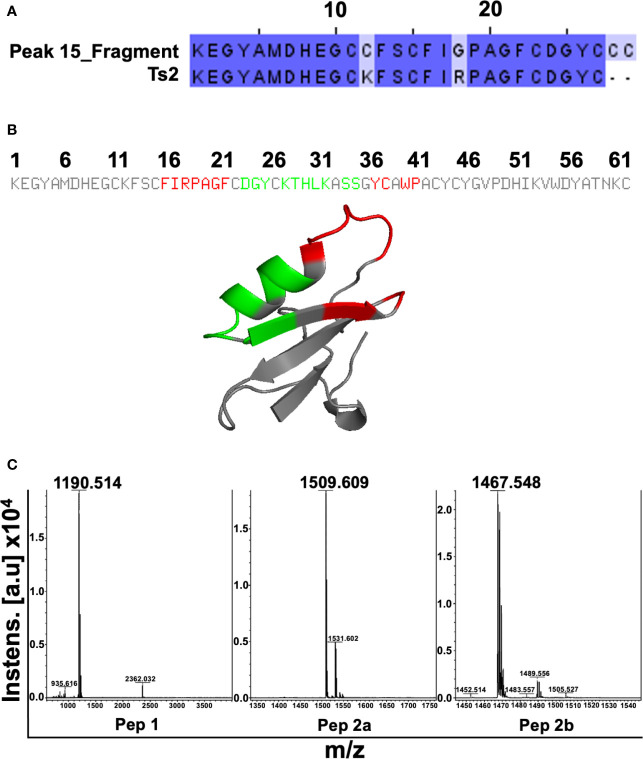
Characterization of Sub6-B subfraction and mimetic peptide production. In order to characterize the molecule (peptide) present in Sub6-B, it was sequenced by Edman degradation, elucidating its partial amino acid sequence: KEGYAMDHEGCCFSCFIGPAGFCDGYCCC…. The next step was to compare this sequence with other sequences available on the online public data bank (BLAST). Sub6-B (Peak_15) sequence had a high (93%) identity with *T. serrulatus* Ts2 toxin (showing conserved amino acids in dark blue and different amino acids in light blue, of which, 27 out of 29 aa of the fragment aligned with Ts2 toxin) **(A)**. Primary and tertiary structures of conformation peptides were designed based on the Ts2 structure. Peptide 1 is highlighted in red and Peptide 2 in green **(B)**. The purity of Pep1, Pep2a, and Pep2b was analyzed using MALDI-TOF-TOF mass spectrometry **(C)**.

To characterize the molecule/peptide present in Sub6-B, peak 15 of this subfraction was sequenced and its partial amino acid sequence was elucidated: KEGYAMDHEGCCFSCFIGPAG FCDGYCCC(…). This amino acid sequence was searched against the NCBI nr protein databases using BLASTp. The highest BLASTp hit was Ts2 toxin (found in TsV – UniProt ID: P68410 [SCX2_TITSE]), with 93% similarity (of note, 27 out of 29 amino acids of the fragment aligned with Ts2 toxin) (**Figure 4A**). As the identified sequence is composed of several amino acids, including cysteine, which is prone to disulfide bond formation, the synthesis of the entire segment might be rather challenging and costly. Therefore, we used the PEPOP tool to design two discontinuous peptides mimicking the Ts2 toxin. The regions from Ts2 used to generate the peptides are highlighted in red and green (**Figure 4B**): Pep1: (*CH_3_CO*- DAYKTHLKSS-*NH_2_*) and Pep2a (*CH_3_CO*-FIRPAGFKYSWP-*NH_2_*), whereas an extra third compound Pep2b (FIRPAGFKYSWP-*NH_2_*) was also proposed by the N-terminal deacetylation of Pep2a. These three peptides were obtained using chemical synthesis, purified by reversed-phase HPLC, and their purity was determined using mass spectrometry. **Figure 4C** depicts representative MS spectra of the synthetized peptides. Observed molecular masses (MS) and amino acid sequences obtained by peptide fragmentation (MS/MS) are in accordance with the expected theoretical values.

### Pep1 and Pep2a, but Not Pep2b, Induce Potent Anti-Protozoal Activity *In Vitro* and Improved the Control of *T. gondii* Infection in Mice

**Figure 5:**
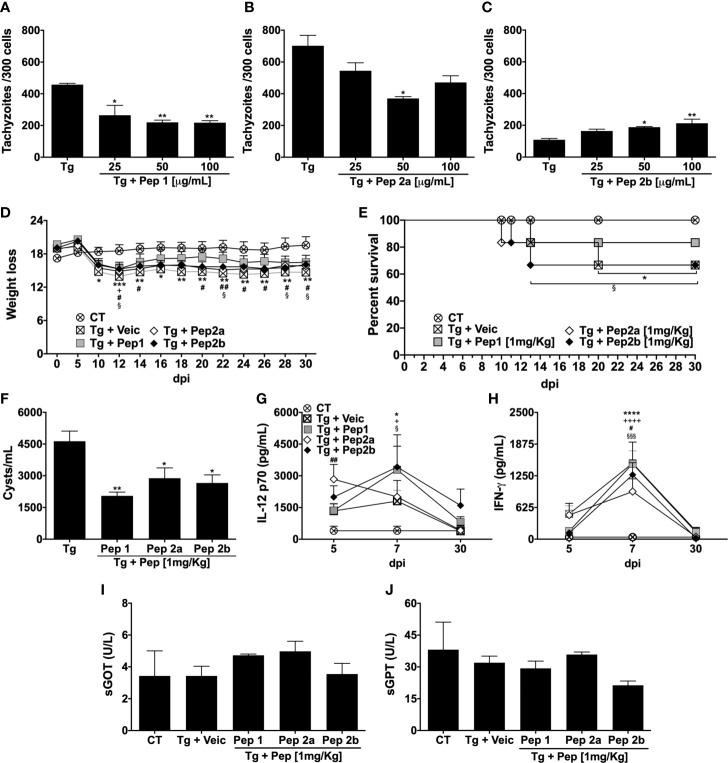
Evaluation of the anti-*T. gondii* activity of Pep1, Pep2a, and Pep2b peptides *in vitro* and *in vivo*. Peritoneal macrophages from C57Bl/6 female mice were cultured and infected (1:1 *T. gondii*: cell ratio) and/or stimulated by Pep1 **(A)**, Pep2a **(B)**, or Pep2b **(C)** at 25, 50, or 100 µg/mL. Intracellular parasite counts were performed on fixed and stained cells at 48 h post-infection/stimulus (Each point represents means ± SEM of one of the two independent experiments, *p ≤ 0.05; **p < 0.01). C57Bl/6 mice (n= 6/group) were infected with 20 cysts of *T. gondii* (ME49 strain) and treated or not with Pep1, Pep2a, or Pep2b (1 mg/kg) at 8 h after infection until the 7^th^ day post infection (dpi). The weight loss **(D)** and survival **(E)** were monitored from the 5^th^–30^th^ dpi. The cytokines IL-12p70 **(G)** and IFN-γ **(H)** were quantified in the serum of the animals on 5^th^, 7^th^, and 30^th^ dpi. The mice were euthanized on 30^th^ dpi for the brain cyst count **(F)** and measurement of liver enzymes in the serum [sGOT **(I)** and sGPT **(J)**]. “CT” = control (non-infected mice). “Tg + Veic”= infected-mice vehicle-injected➔ *p ≤ 0.05; **p< 0.01; ***p < 0.001; ****p < 0.0001 (CT x Tg + Veic)→ ^+^ *p* ≤ 0.05; ^+++^ *p* < 0.001; ^++++^ *p* < 0.0001 (CT x Tg + Pep1)→ ^#^ *p* ≤ 0.05; ^##^ *p* < 0.01 (CT x Tg + Pep2a)→ ^§^ *p* ≤ 0.05; ^§§§^ *p* < 0.001 (CT x Tg + Pep2b). Each point represents means ± SEM of two independent experiments.

To determine whether the synthetic peptides could control parasite replication, MOs were infected and stimulated with Pep1, Pep2a, or Pep2b at concentrations of 100, 50, or 25 µg/mL. Pep1 at all concentrations (**Figure 5A**) and Pep2a at 50 µg/mL (**Figure 5B**) reduced the intracellular replication of tachyzoites compared with the infected non-stimulated cells. In contrast, stimulation with Pep2b at 50 or 100 µg/mL resulted in increased parasite replication compared to the unstimulated infected cells (**Figure 5C**). The antiparasitic activity of the peptides was also evaluated *in vivo* by *T. gondii*-infected mice. Our results showed that the treatment of mice with Pep1 reversed from the 14^th^ dpi, the weight loss caused by the infection. In contrast, untreated-infected animals lost weight from the 5^th^–30^th^ dpi (end of the study) (**Figure 5D**). To a lesser extent, peptides 2a and 2b also reduced the weight loss caused by *T. gondii* infection (**Figure 5D**). The ability of Pep1 and Pep2a to reduce weight loss correlated with a higher survival rate in the group of animals treated with these peptides (**Figure 5E**). Despite some differences in weight loss and survival, curiously, all peptides tested were able to reduce the number of brain cysts in infected mice at 30^th^ dpi (**Figure 5F**), emphasizing the best ability of Pep1 to improve all the analyzed parameters. In addition, our results suggest that the protection (reduction in the number of cysts and/or higher survival rate) offered by Pep1, Pep2a, or Pep2b treatment was independent of the systemic production of cytokines IL-12 (**Figure 5G**) or IFN-γ (**Figure 5H**), since all treated groups presented similar levels of these cytokines correlating to the untreated-infected group at all analyzed time points. Importantly, the analysis of liver enzymes present in the serum (GOT; **Figure 5I** and GPT; **Figure 5J**) at 30^th^ dpi revealed that the treatment of animals with these peptides did not induce late hepatotoxicity.

## Discussion

Current therapies for treating toxoplasmosis are not yet effective in eradicating the parasite from the host and present toxic side effects. The ability of TsV to activate immune cells (Petricevich et al., 2008; Zoccal et al., 2011; Casella-Martins et al., 2015; Pimentel et al., 2021) allowed us to hypothesize that its compounds could act as anti-toxoplasma agents. Herein, MOs were highlighted owing to their anti-protozoal capacity during parasite infection (Stafford et al., 2002; Pimentel et al., 2021).

We demonstrated that crude TsV and its F6 and F7 fractions induced NO production by MOs without causing host cell toxicity. It was of great relevance since NO is a potent microbicidal/microbiostatic agent controlling *T. gondii* replication (Seabra et al., 2002) and corroborated previous studies using cell lines (Zoccal et al., 2011) and murine macrophage (Pimentel et al., 2021). Moreover, despite the well-known ability of *T. gondii* to suppress or evade host immunity, TsV significantly increased NO production by controlling *T. gondii* replication.

TsV was described to stimulate MOs through a complex interaction involving membrane receptors (e.g., Toll-like receptor 2/4) (Zoccal et al., 2014), ion channels (e.g., Na^+^, Ca^+^, and K^+^) (Lowry et al., 1998; Oliveira et al., 2019), and Mitogen-Activated Protein Kinases (MAPK) (Pimentel et al., 2021) which culminate in the production of inflammatory mediators. Cytokines play a key role in amplifying the response/communication among cell types, building an efficient response against pathogens. Herein, TsV, F6, and F7 fractions were potent inducers of IL-12p70/TNF/IL-6 in MOs, which is consistent with previous studies demonstrating that TsV-stimulated MOs secrete IL-1/IL-6/TNF/IFN-γ (Petricevich, 2002; Oliveira et al., 2019; Pimentel et al., 2021). Of great relevance, we demonstrated that TsV compounds induced the production of IL-12 and TNF by *T. gondii*-infected MOs, abolishing the capacity of the parasite to evade host immunity. The ability of this parasite may be higher or lower owing to the *T. gondii* strain and protein secretion from specialized organelles such as micronemes, rhoptries, and dense granules (Melo et al., 2011; Hunter and Sibley, 2012).

Hitherto, our data led us to hypothesize that TsV and F6 were able to reduce tachyzoite replication in MOs, activating a pro-inflammatory response. Reinforcing this hypothesis, pre-stimulation of the tachyzoites with TsV or F6 before infection did not alter the replicative capacity of the parasite, excluding a direct action on the parasite. Unlike TsV, the F6 fraction was able to stimulate tachyzoite invasion in MOs. We speculate that this was due to the modifications on the membrane of the host cell, facilitating the parasite invasion and/or by activation of the host receptors that sense parasite factors, favoring parasite internalization (Bonhomme et al., 1999; Sweeney et al., 2010).

Because of the sustained ability of Sub6-B to control both parasite replication and immune response, the partial amino acid sequence that makes up this toxin was identified and compared using BLASTp. The Sub6-B partial sequence has 93% similarity with the Ts2 toxin sequence, suggesting that this molecule could be the Ts2 toxin or has high homology to it. Ts2 has been described as having 72% identity with Ts1 (Mansuelle et al., 1992), being able to prolong the duration of the action potential of rabbit vagus-nerve, as an α-toxin that inhibits the inactivation of some Na^+^ channels (e.g., NaV1.2/NaV1.3/NaV1.5/NaV1.6) (Cologna et al., 2012), and presents immunomodulatory actions (Zoccal et al., 2011). Ts2 was demonstrated to induce pro- and anti-inflammatory profiles *in vivo* (Zoccal et al., 2013). Ts2-intraperitoneal injection-induced recruitment of leukocytes (e.g., neutrophils, mononuclear cells, CD4, and CD8 lymphocytes) associated with increased production of pro-inflammatory (IL-6/TNF/IFN-γ/IL-1β/leukotriene-B4/prostaglandin-E2) and anti-inflammatory/pro-resolving (IL-10/IL-4) mediators. Thus, our results suggest that Sub6-B increased anti-*T. gondii* ability, inducing the production of inflammatory molecules.

To optimize peptide production using chemical synthesis, two Ts2 toxin mimetic peptides were designed using the PEPOP program. After *in silico* analysis, we selected and combined some amino acid sequences from the Ts2 toxin that gave rise to two peptides, namely Pep1 and Pep2a as well as the derivative Pep2a sequence, Pep2b, which carries no acetylation at its N-terminus. These peptides are approximately five times smaller than the original sequence and are easily obtained by solid-phase synthesis. The predicted activity of peptides *in silico* was checked using both *in vitro* and *in vivo* models of *T. gondii* infection. Pep1 and Pep2a stimulation reduced parasite replication in macrophages, whereas Pep2b improved it. By adding an acetyl group at the N-terminal amino acid of a peptide, it is possible to change some of its physico-chemical properties such as hydrophobicity and charge distribution (by quenching the N-terminus positive charge). As a result, some structural or biochemical features of the original peptide might be affected, including (but not restricted to) folding, hydrolysis prevention, protein interactions, and cell permeation or subcellular location (reviewed by Ree et al., 2018). Although any of the above-mentioned explanations (or even a sum of them), could explain the different activities between Pep2a and Pep2b, further studies are needed to better understand this interesting finding. *In vivo*, although only Pep1 improved the tested clinical parameters of the disease, the treatment with all peptides showed decreases in cerebral parasitism and did not induce chronic/late liver injury. Such discrepant effects of Pep2b on parasite growth, *in vitro* and *in vivo*, may be explained by the fact that only MOs were stimulated *in vitro*, and in the *in vivo* system, the peptides may activate different cell types that act cooperatively to reduce parasite replication/dissemination. Moreover, differences between *T. gondii* strains used *in vitro* (RH strain) and *in vivo* (ME49 strain) should also be considered (Hwang et al., 2018). Here, the anti-*T. gondii* therapeutic effect of the peptides was independent of IL-12 and IFN-γ systemic levels. However, the participation of IL-12 and IFN-γ cannot be totally excluded, since the peptides may reach specific cells, inducing the production of these cytokines in an organ-specific manner, such as in the lymphoid and brain, thereby reducing parasite load.

## Conclusion

Collectively, our results reveal for the first time that molecules from *T. serrulatus* venom could have promising potential as therapeutic agents in toxoplasmosis. Of great relevance, we demonstrate that synthetic peptides treatment *in vivo* reduces the number of brain cysts number without inducing hepatotoxicity, indicating a highly required characteristic for molecules to be used in the treatment of toxoplasmosis: acting at the focal point of the infection, mainly in the central nervous system.

## Data Availability Statement

The raw data supporting the conclusions of this article will be made available by the authors, without undue reservation.

## Ethics Statement

The animal study was reviewed and approved by The UFMG Animal Ethics Committee (CEUA) approved all experiments and procedures (Permit Number 449/2015).

## Author Contributions

DA, ML, AP, MB, and FM conceived experiments. DA, MT, ML, AP, RV, JR, LF, CO, and FM wrote the manuscript. DA, PP, PR, RR, LF, JR, MB, and MC performed experiments. LF, CO, RV, MB, ML, AP, MT, ML, AP, and FM supervised and provided expertise and funding. All authors contributed to the article and approved the submitted version.

## Funding

This work was supported by Pró-Reitoria de Pesquisa/UFMG, Conselho Nacional de Desenvolvimento Científico e Tecnológico (CNPq: 305894/2018-8; 438054/2018-0) and Fundação de Amparo a Pesquisa de Minas Gerais (FAPEMIG: APQ-02331-18; Rede Mineira de Imunobiológicos, REDE-00140-16), Coordenação de Aperfeiçoamento de Pessoal de Nível Superior (CAPES: CAPES/COFECUB 57914) (Brazil) and the National Institute for Science and Technology in Dengue and Host-microbial interactions (465425/2014-3). DA, PP, PR, RR, RV, LF, CO, JR, MT, AP, ML, and FM acknowledge grants from CNPq.

## Conflict of Interest

The authors declare that the research was conducted in the absence of any commercial or financial relationships that could be construed as a potential conflict of interest.
